# Role of Integrin β4 in Lung Endothelial Cell Inflammatory Responses to Mechanical Stress

**DOI:** 10.1038/srep16529

**Published:** 2015-11-17

**Authors:** Weiguo Chen, Yulia Epshtein, Xiuquin Ni, Randal O. Dull, Anne E. Cress, Joe G.N. Garcia, Jeffrey R. Jacobson

**Affiliations:** 1 Division of Pulmonary, Critical Care, Sleep, and Allergy, University of Illinois at Chicago, Chicago, IL; 2 Department of Anatomy, Harbin Medical University at Daqing, China; 3 Department of Anesthesiology, University of Illinois at Chicago, Chicago, IL; 4 Department of Medicine, University of Arizona, Tucson, AZ

## Abstract

Simvastatin, an HMG-CoA reductase inhibitor, has lung vascular-protective effects that are associated with decreased agonist-induced integrin β4 (ITGB4) tyrosine phosphorylation. Accordingly, we hypothesized that endothelial cell (EC) protection by simvastatin is dependent on these effects and sought to further characterize the functional role of ITGB4 as a mediator of EC protection in the setting of excessive mechanical stretch at levels relevant to ventilator-induced lung injury (VILI). Initially, early ITGB4 tyrosine phosphorylation was confirmed in human pulmonary artery EC subjected to excessive cyclic stretch (18% CS). EC overexpression of mutant ITGB4 with specific tyrosines mutated to phenylalanine (Y1440, Y1526 Y1640, or Y1422) resulted in significantly attenuated CS-induced cytokine expression (IL6, IL-8, MCP-1, and RANTES). In addition, EC overexpression of ITGB4 constructs with specific structural deletions also resulted in significantly attenuated CS-induced inflammatory cytokine expression compared to overexpression of wildtype ITGB4. Finally, mice expressing a mutant ITGB4 lacking a cytoplasmic signaling domain were found to have attenuated lung injury after VILI-challenge (V_T_ = 40 ml/kg, 4 h). Our results provide mechanistic insights into the anti-inflammatory properties of statins and may ultimately lead to novel strategies targeted at ITGB4 signaling to treat VILI.

Acute lung injury (ALI) is a challenging clinical problem characterized by increased lung vascular permeability and inflammation and associated with significant morbidity and mortality^1^. Clinically, ALI can be precipitated or exacerbated by excessive lung stretch associated with mechanical ventilation, a condition known as ventilator-induced lung injury (VILI). However, aside from the use of low tidal volume mechanical ventilation which was first reported over a decade ago^2^, there are no therapies currently in practice that are able to definitively improve outcomes in patients with ALI/VILI. We previously reported the protective effects of simvastatin, an HMG coA-reductase inhibitor, in a murine model of LPS-induced ALI^3^. The mechanisms underlying the protective effects of simvastatin in this context are complex and involve differential effects on endothelial cell (EC) Rho GTPases^4^,^5^, actin cytoskeletal rearrangement^4^, and NADPH oxidase inhibition^5^ as well as differential EC gene expression including the upregulation of the tight junctional protein, claudin-5^6^. While these effects all contribute to murine ALI protection by statins, we identified integrin β4 (ITGB4) as the most upregulated gene in EC treated with simvastatin^4^ and confirmed that ITGB4 is a critical mediator of EC inflammatory responses relevant to ALI, evidenced by the abrogation of murine ALI protection by simvastatin in response to the use of an ITGB4 blocking antibody^7^. Subsequently, we have begun to characterize the role of ITGB4 in normal lung EC signaling and function^8^,^9^ and have identified ITGB4-mediated vascular inflammatory responses. In this study we sought to further define functional role of ITGB4 in lung EC inflammatory responses mediated by excessive mechanical stress consistent with levels able to precipitate VILI.

The integrins, expressed on a variety of cell types, are transmembrane heterodimers consisting of α and β subunits that mediate both inside-out and outside-in signaling. While the role of ITGB4 in ALI is not well characterized, a growing literature has implicated other β integrins as effectors of lung vascular permeability and EC barrier function^10^,^11^,^12^. For example, ITGB5 has been identified as a mediator of ALI in separate models of rat ischemia-reperfusion lung injury and murine ALI^10^. Moreover, ITGB5 inhibition *in vitro* was found to attenuate agonist-induced EC actin stress fiber formation and monolayer permeability. Additionally, in separate reports, ITGB2^12^, ITGB3^13^ and ITGB6^11^ have also been implicated as mediators of lung vascular permeability associated with ALI, although the precise mechanisms of integrin-mediated effects in these contexts remain to be fully characterized.

While 8 β subunits have been identified, the laminin-5 receptor ITGB4, which forms a heterodimer only with integrin α6, is uniquely characterized by its long cytoplasmic tail of over 1000 amino acids. This tail is comprised of a proximal Calx Na-Ca exchanger domain followed by two pairs of fibronectin type III repeats separated by a tyrosine activation motif (TAM)^14^. The extracellular portion of the β4 subunit sequence is found to have 35% homology with other integrin β subunits but is the most different within this class of molecules. The transmembrane region is poorly conserved, whereas the cytoplasmic domain shows no substantial identity in any region to the cytoplasmic tails of the known β sequences or to other protein sequences. The exceptionally long cytoplasmic domain of ITGB4 suggests potentially distinct protein interactions and downstream signaling relative to the other β integrins. In this study we utilized various ITGB4 constructs with specific mutations or deletions within the cytoplasmic tail to characterize its role in the elaboration of inflammatory responses of mechanically stressed lung EC *in vitro*. In separate experiments, we subjected mice expressing a mutant ITGB4 lacking a cytoplasmic signaling domain (terminating at amino acid 1355) to VILI and assessed lung vascular leak and inflammation. Collectively, these studies further define the functional role of EC ITGB4 as a mediator of ALI/VILI and suggest selective targeting of ITGB4 signaling may represent a novel therapeutic strategy clinically.

## Results

### ITGB4 phosphorylation in response to mechanical stress

**Figure 1:**
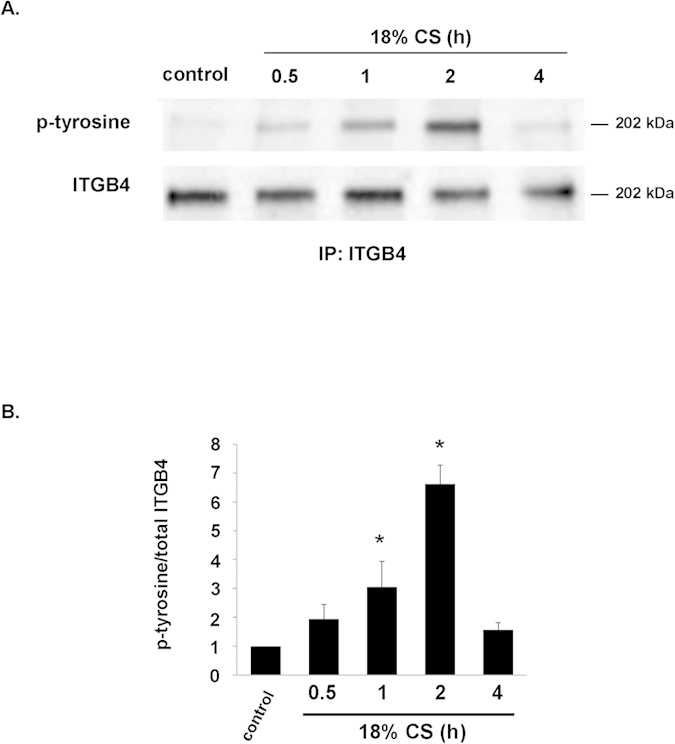
Mechanical stress of ITGB4 phosphorylation in human lung EC. (**A**) Human pulmonary artery EC were grown to confluence on Bioflex plates and then subjected to 18% CS (0–4 h). Cell lysates were then used for immunoprecipitation (IP) using an anti-ITGB4 antibody followed by Western blotting for phosphorylated tyrosine (p-tyrosine; representative blots shown). (**B**) Results of densitometry expressed as p-tyrosine/total ITGB4 are shown (n = 3/condition, *p < 0.05 compared to control).

In initial experiments, human pulmonary artery EC were grown to confluence on Bioflex plates and then subjected to 18% cyclic stretch (CS) for up to 4 h prior to collection of cell lysates for immunoprecipitation of ITGB4 followed by Western blotting for p-tyrosine (Fig. 1). These studies confirmed increased tyrosine phosphorylation of ITGB4 in response to CS evident within 30 min with peak phosphorylation at 2 h. These data are consistent with our previous report of increased EC ITGB4 phosphorylation in response to lipopolysaccharide (LPS)^7^, as both LPS and excessive mechanical stress induce EC barrier disruption *in vitro* and inflammatory lung injury *in vivo*.

### Role ITGB4 tyrosine phosphorylation in EC inflammatory responses to cyclic stretch

**Figure 2:**
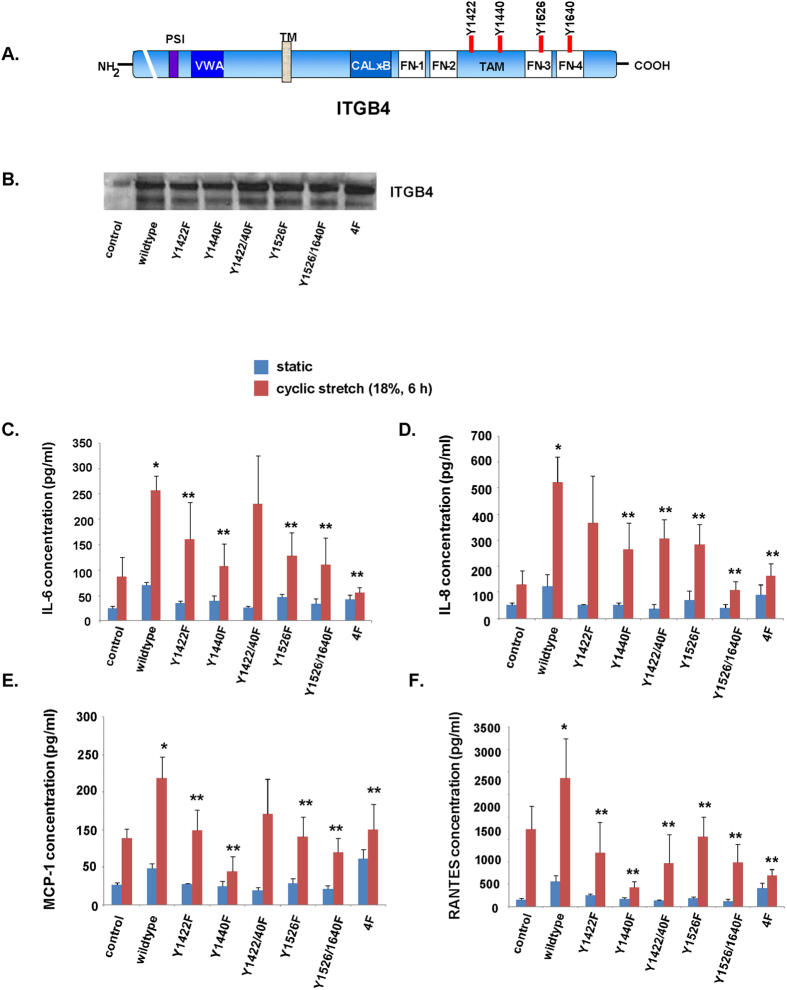
ITGB4 tyrosine phosphorylation mediates CS-induced EC inflammatory responses. Human pulmonary artery EC were transfected with vector (control), wildtype ITGB4 or specific constructs in which individual tyrosine residues (Y) were mutated to phenylalanine (**F**, panel **A**) prior to being subjected to excessive mechanical stretch (18% CS, 6 h). (**B**) Whole cell lysates were used for Western blotting and were probed for ITGB4 to confirm overexpression. (**C**–**F**) Inflammatory cytokines IL-6, IL-8, MCP-1 and RANTES were measured in the media after CS as shown (n = 3/condition, *p < 0.05 compared to static wildtype controls, **p < 0.05 compared to wildtype cells subjected to CS). (TM = transmembrane domain, PSI = plexin-semaphorin-integrin domain, VWA = von Willebrand factor A domain, TM = transmembrane domain, FN = fibronectin type III domain, TAM = tyrosine activation motif).

As a model of VILI *in vitro* we utilized a Flexcell Strain Unit (FX-3000; FlexCell International) and subjected human pulmonary artery EC grown on Bioflex plates to 18% cyclic stretch (CS) as we have previously described^15^. This level of stretch is consistent with pathologic lung stretch that occurs clinically in the setting of VILI while 5% CS corresponds to physiologic changes associated with normal respiration in the healthy lung^15^. To investigate the role of ITGB4 tyrosine phosphorylation in the expression of inflammatory cytokines induced by the mechanical stress of lung EC we first utilized a series of six ITGB4 constructs in which one of four tyrosines (Y1440, Y1526, Y1640, or Y1422) were mutated to phenyalanine (F), either individually or in combination (Fig. 2A). Initially, we confirmed effective overexpression by Western blotting of whole cell lysates (Fig. 2B). These blots demonstrated two distinct bands upon probing for ITGB4 which we speculate may be due to either variable posttranslational modifications such as glycosolation^16^ or proteolysis of ITGB4^17^. Subsequently, in all but three cases, transfection of EC with an ITGB4 mutant resulted in a significant attenuation of the expression of inflammatory cytokines IL-6, IL-8, MCP-1 or RANTES in the media after 18% CS (6 h) compared to cells overexpressing wildtype ITGB4 (Fig. 2C–F). Interestingly, two of the three assays that did not demonstrate a significant effect involved constructs in which Y1422 and Y1440 were both mutated suggesting that this combined mutation has effects on EC inflammatory signaling induced by mechanical stress that effectively counteracts the predominant anti-inflammatory effects associated with the mutation of either one of these tyrosines by itself. There was no appreciable effect of overexpression of either wildtype ITGB4 or any of the mutants on cytokine levels detectable in the media of static cells.

### Role ITGB4 cytoplasmic domain in EC inflammatory responses to cyclic stretch

**Figure 3:**
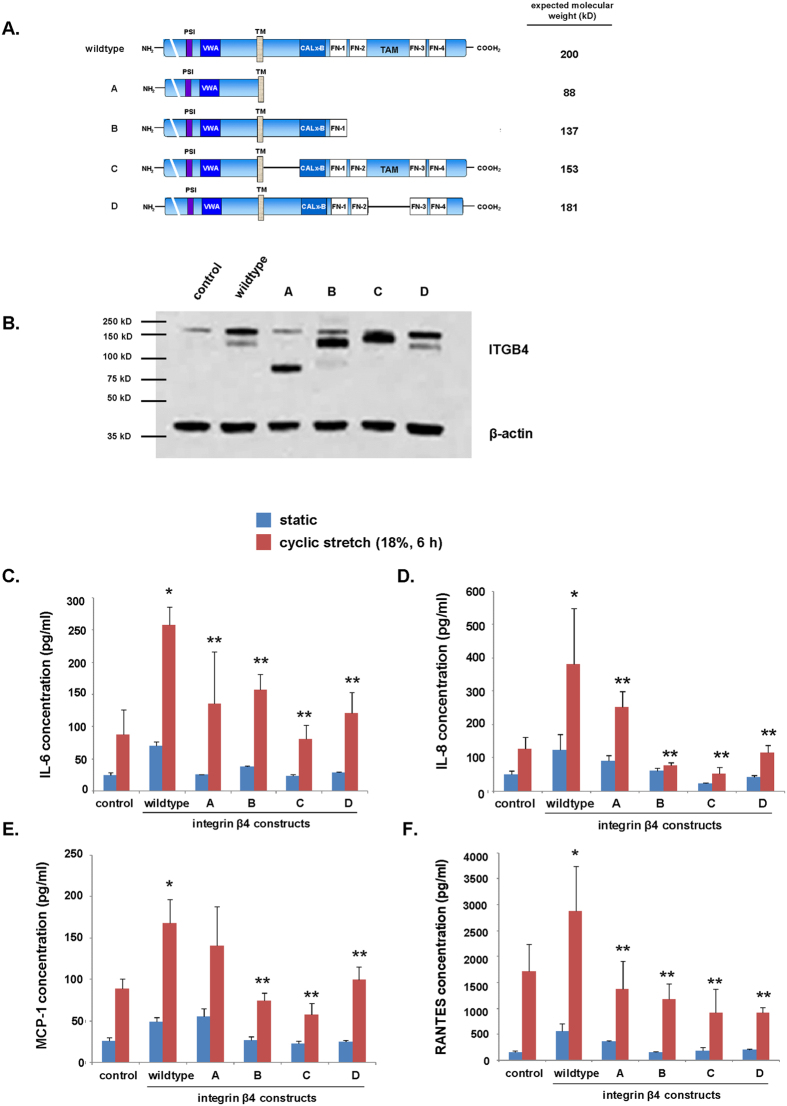
ITGB4 cytoplasmic domains mediate cyclic CS-induced EC inflammatory responses. Human pulmonary artery EC were transfected with wildtype ITGB4 or specific deletion constructs (**A**) prior to being subjected to excessive mechanical stretch (18% CS, 6 h). (**B**) Whole cell lysates were used for Western blotting and were probed for ITGB4 to confirm overexpression. (**C**–**F**) IL-6, IL-8, MCP-1 and RANTES were measured in the media after CS as shown (n = 3/condition, *p < 0.05 compared to static wildtype controls, **p < 0.05 compared to wildtype cells subjected to CS). (TM = transmembrane domain, PSI = plexin-semaphorin-integrin domain, VWA = von Willebrand factor A domain, TM = transmembrane domain, FN = fibronectin type III domain, TAM = tyrosine activation motif).

We conducted identical experiments using a separate series of ITGB4 constructs in which specific cytoplasmic domains were deleted (Fig. 3A). Western blotting again confirmed overexpression of constructs with bands noted at variable molecular weights corresponding to the expression of both endogenous ITGB4 and the individual transfected constructs (Fig. 3B). Compared to cells overexpressing wildtype ITGB4, transfection of EC with any of these ITGB4 mutants resulted in a significant attenuation of the expression of inflammatory cytokines IL-6, IL-8, MCP-1 and RANTES in the media after CS (18%, 6 h), with only one exception noted (Fig. 3C–F). This single outlier was observed with CS-induced MCP-1 expression in cells transfected ITGB4 completely lacking a cytoplasmic domain (A). Conversely, we observed a non-significant trend that transfection with ITGB4 lacking the proximal region of the cytoplasmic domain (C) was associated with the most prominent and consistent effects on the reduced elaboration of inflammatory cytokines after mechanical stress. Consistent with the previous series of experiments, there was again no appreciable effect of overexpression of either wildtype ITGB4 or any of the mutants on cytokine levels detectable in the media of static cells.

### Role of ITGB4 cytoplasmic domain in the elaboration of murine VILI

**Figure 4:**
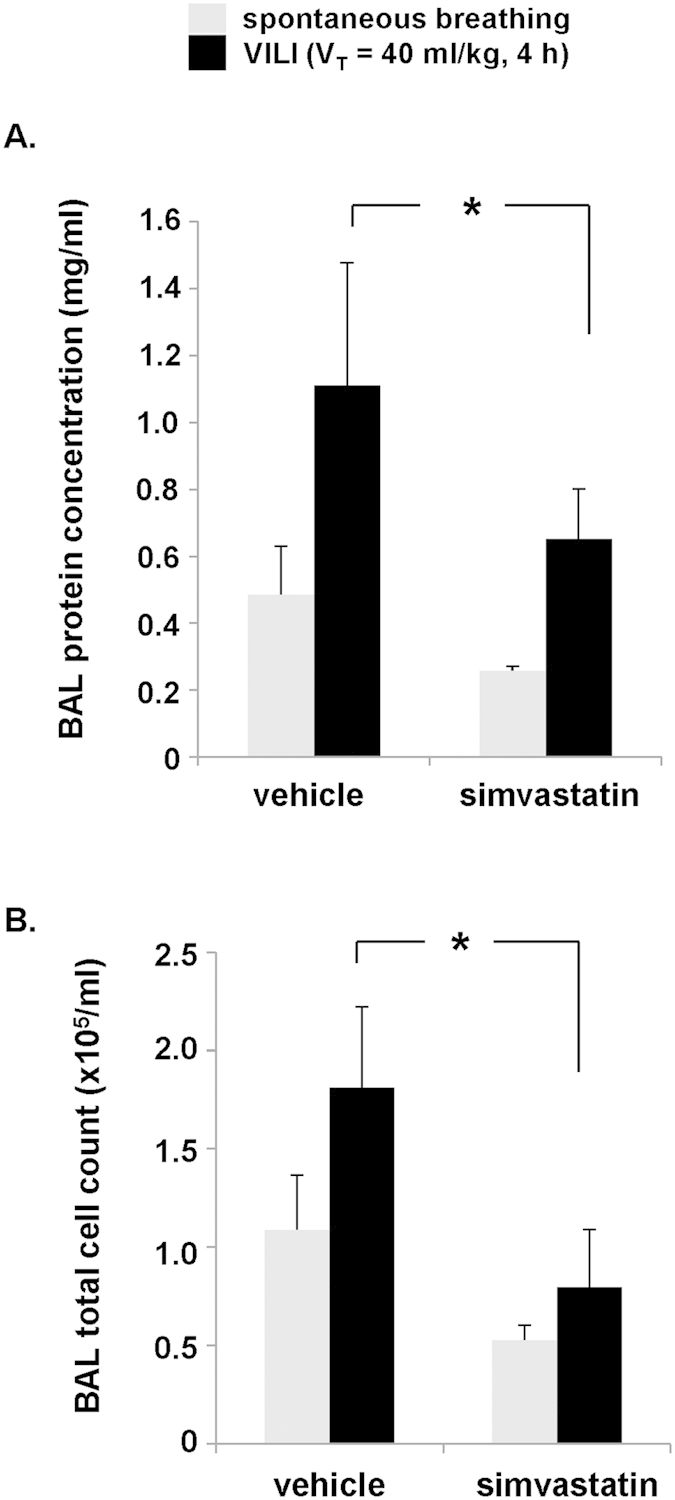
Simvastatin attenuates vascular permeability and inflammation in mice subjected to VILI. Wildtype mice were treated with simvastatin (20 mg/kg, IP injection) or vehicle 16 h prior to being subjected to VILI challenge (V_T_ =  40 ml/kg, 4 h). Spontaneously breathing mice were used as controls. BAL fluid was then collected and analyzed for (**A**) protein content and (**B**) total cell counts (*p < 0.05, n = 3 animals/condition).

To investigate the significance of our results in an *in vivo* VILI model we initially confirmed the protective effects of simvastatin in this context consistent with other reports^18^. Mice were treated with simvastatin (20 mg/kg, IP injection) or vehicle 16 h prior to being subjected to high tidal volume mechanical ventilation (V_T_ = 40 ml/kg, 4 h) to induce VILI. Compared to controls, animals that received simvastatin were found to have significantly reduced bronchoalveolar lavage (BAL) fluid protein levels and total cell counts consistent with an attenuated VILI-associated inflammatory response (Fig. 4).

**Figure 5:**
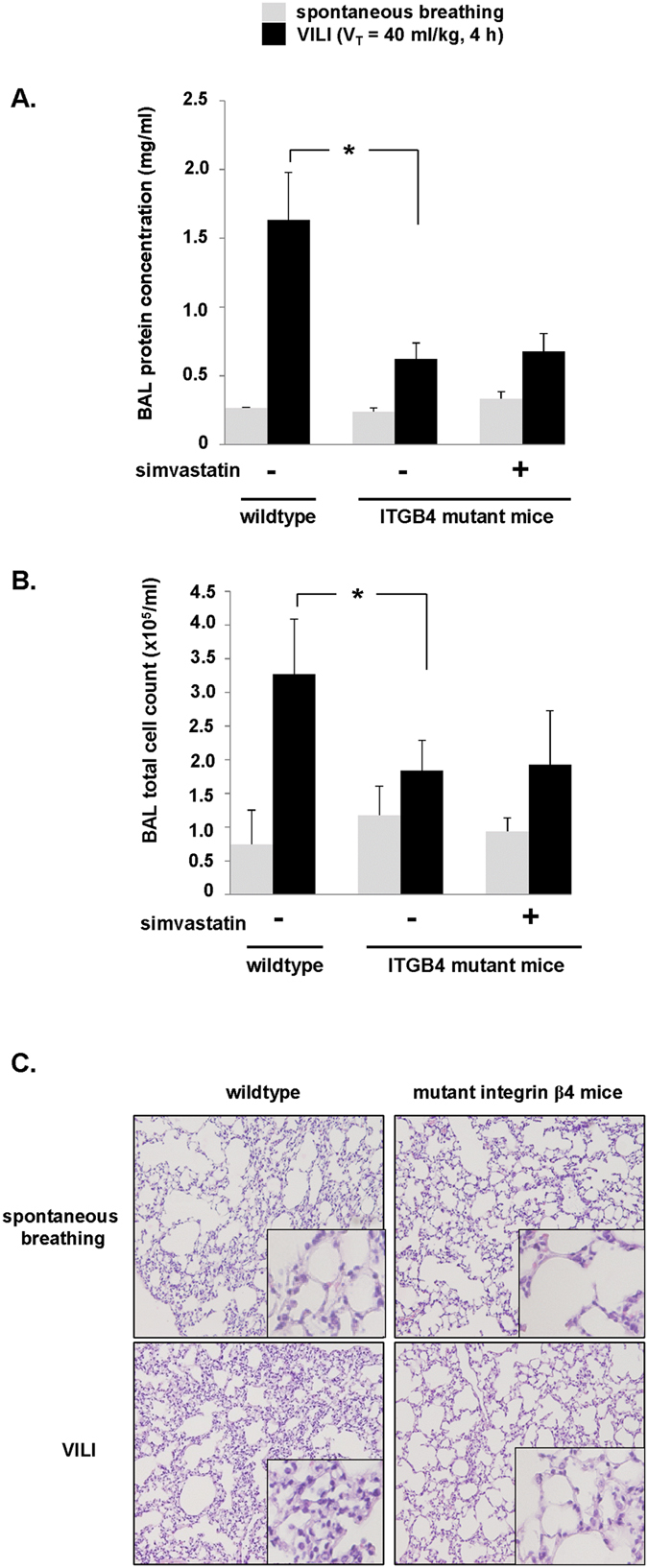
Decreased lung vascular permeability and inflammation in ITGB4 mutant mice subjected to VILI. Wildtype mice and mice expressing a mutant ITGB4 lacking a cytoplasmic signaling domain were pretreated with simvastatin (20 mg/kg, IP injection, 24 h) or vehicle prior to VILI challenge (V_T_ = 40 ml/kg, 4 h). Spontaneously breathing mice were used as controls. BAL fluid was then collected and analyzed for (**A**) protein content and (**B**) total cell counts (*p < 0.05, n = 5 animals/condition). (**C**) In separate experiments, lungs were collected from spontaneously breathing and VILI-challenged wildtype and ITGB4 mutant animals and used for histology. Representative images are shown (10× magnification, insets 40×).

Next, we subjected genetically engineered mice expressing ITGB4 lacking a cytoplasmic signaling domain to high tidal volume mechanical ventilation and assessed indices of lung vascular permeability and inflammation. Compared to wildtype control animals, mutant ITGB4 mice were found to have significantly attenuated inflammatory response to VILI-challenge (V_T_ = 40 ml/kg, 4 h) as assessed by BAL fluid protein and total cell counts (Fig. 5A,B). Notably, while simvastatin was protective in our VILI model, there was no appreciable effect of simvastatin pretreatment (20 mg/kg, IP, 16 h prior to mechanical ventilation) in ITGB4 mutant mice subjected to VILI. These results suggest that protection by simvastatin in our murine VILI model is strongly influenced by its effects on ITGB4.

**Figure 6:**
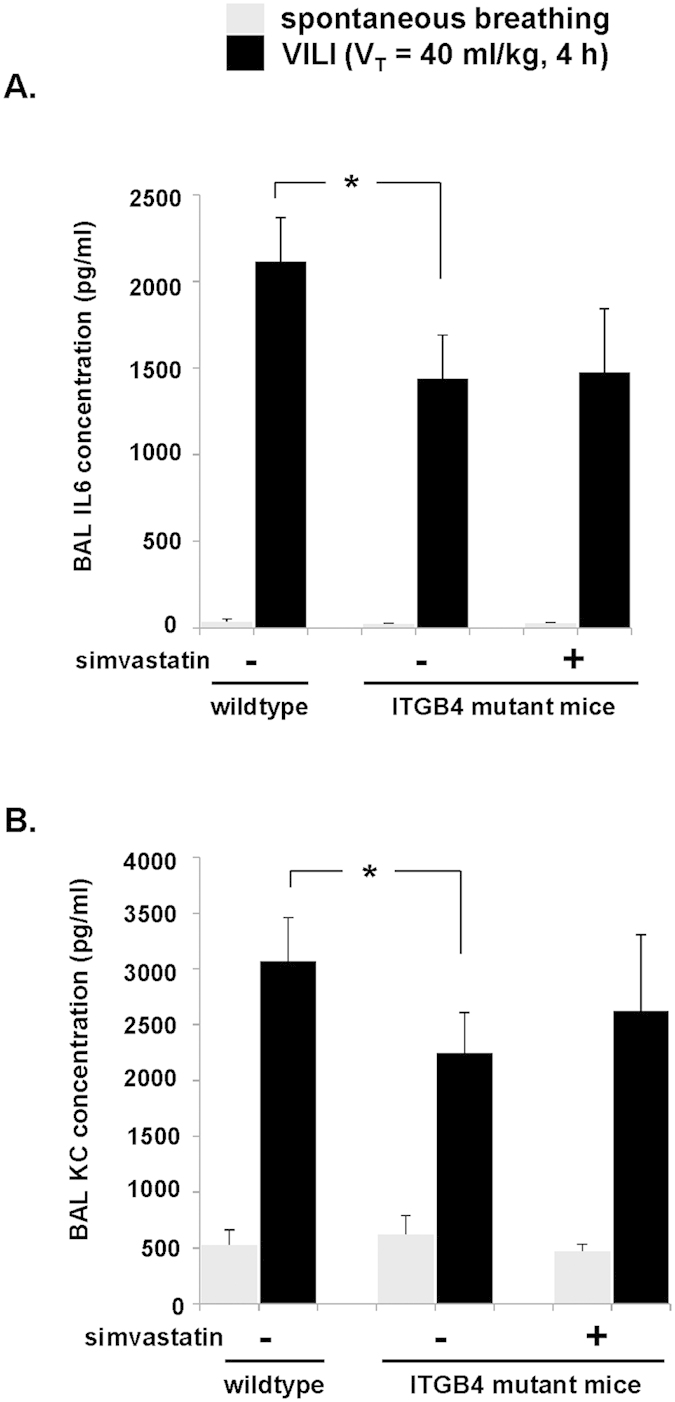
Decreased lung inflammatory cytokines in ITGB4 mutant mice subjected to VILI. Wildtype mice and mice expressing a mutant ITGB4 lacking a cytoplasmic signaling domain were pretreated with simvastatin (20 mg/kg, IP injection, 24 h) or vehicle prior to VILI challenge (V_T_ = 40 ml/kg, 4 h). BAL fluid was then collected and inflammatory cytokine levels measured including (**A**) IL-6 and (**B**) KC. (*p < 0.05, n = 5 animals/condition).

In the same experiments lung histology demonstrated infiltration of inflammatory cell and edema in wildtype mice in response to VILI that was markedly reduced in mutant ITGB4 mice after VILI (Fig. 5C). Finally, BAL fluid collected for measurement of inflammatory cytokines, IL-6 and KC (a murine IL-8 homolog), confirmed significantly lower levels of both cytokines in the BAL fluid of ITGB4 mutant mice subjected to VILI compared to VILI-challenged controls (Fig. 6). Similarly, there was no effect of simvastatin pretreatment on IL-6 and KC levels in ITGB4 mutant mice after VILI.

## Discussion

In this study we confirmed the functional importance of ITGB4 in the elaboration of lung EC inflammatory responses to mechanical stress both *in vitro* and *in vivo*. Specifically, we observed increased tyrosine phosphorylation of ITGB4 in human pulmonary artery EC subjected to excessive CS while the overexpression of full length ITGB4 was associated with increased CS-induced EC expression of inflammatory cytokines. In contrast, overexpression of any one of several distinct ITGB4 constructs harboring mutations in the cytoplasmic tail, defined broadly by either mutations of tyrosine phosphorylation sites or deletion of structural domains, resulted in decreased mechanical stress-induced inflammatory cytokines released by EC. Moreover, mice expressing ITGB4 lacking a cytoplasmic signaling domain altogether were protected from VILI compared to VILI-challenged wildtype animals. These results are consistent with our hypothesis that phosphorylated ITGB4 is pro-inflammatory and, conversely, a relative decrease in phosphorylated ITGB4 is anti-inflammatory. These findings further support emerging evidence for the critical role of ITGB4 in the elaboration of ALI/VILI.

The functional role of ITGB4 tyrosine phosphorylation has previously been studied in a variety of other contexts. For example, the binding of the adaptor protein, Shc, to ITGB4 is dependent on phosphorylation of Y^1140^ and Y^1526^ and is associated with both downstream MAPK signaling and inhibition of hemidesmosome formation in epithelial cells^19^. More relevant to the current study, we previously reported that inhibition of ITGB4 tyrosine phosphorylation is associated with an attenuation of EC barrier augmentation by hepatocyte growth factor^20^, an agonist that is also protective in murine ALI^21^. We have also previously identified ITGB4 as a robust mediator of murine ALI/VILI protection by statins associated with potent anti-inflammatory effects. Specifically, simvastatin induces increased expression of ITGB4 in lung EC while the protective effects of simvastatin in murine ALI can be mitigated by pretreatment with an ITGB4 blocking antibody^7^. The dramatic upregulation of ITGB4 by simvastatin is associated with decreased agonist-induced tyrosine phosphorylation of ITGB4 which in turn attenuates MAPK signaling and downstream expression of inflammatory mediators, including IL-6 and IL-8^7^. Thus, our previous findings suggest that increased expression of ITGB4 harboring unphosphorylated tyrosines within its cytoplasmic tail effectively serves as a “brake” on lung EC inflammatory responses. Our current data extend our prior observations and confirm a critical role for ITGB4 in EC barrier regulation with complex effects dependent on the specific context and support the idea that increased tyrosine phosphorylation is requisite for the pro-inflammatory effects of ITGB4.

While we did not investigate the events responsible for the attenuation of ITGB4 tyrosine phosphorylation by simvastatin one potential mechanism is the inhibition of specific Src family kinases (SFKs), including Fyn and/or Yes, that are known to mediate these events^22^. The downstream consequences of these effects may include the attenuation of Shc and Shp-2 recruitment resulting in decreased Ras and Erk signaling^19^,^23^. Consistent with this, we previously reported decreased agonist-induced Shp-2 phosphorylation and Erk activation in EC expressing decreased ITGB4 (via silencing RNA)^8^. In addition, although our studies focused on the role of ITGB4 tyrosine phosphorylation, three of the constructs studied (constructs A, B, and D; Fig. 3A) were also characterized by the deletion of serines (S1356, S1360, and S1364) that are known to drive ITGB4 signaling upon phosphorylation via epidermal growth factor (EGF) and/or protein kinase C-α (PKCα)^24^.

Several other questions remain to be explored regarding EC inflammatory signaling by ITGB4. Among these are the mechanisms by which overexpression of ITGB4 lacking a proximal cytoplasmic domain, but with otherwise preserved tyrosine phosphorylation sites (construct C, Fig. 3A), is associated with reduced inflammatory responses as we observed in mechanically-stressed EC. Notably, as has been previously reported, this construct is associated with differential effects on ITGB4 phosphorylation compared to wildtype ITGB4 which may account for this observation^19^. Additionally, our results indicate anti-inflammatory effects associated with the overexpression of ITGB4 lacking a cytoplasmic tail altogether (construct A) that were less robust than that observed with each of the constructs with only partial deletions of the cytoplasmic tail (B–D). While further study is needed to fully understand the mechanisms underlying this observation, it is possible that the ITGB4 cytoplasmic tail harbors both pro- and anti-inflammatory regions and the constructs with only partial deletions may actually have a net anti-inflammatory effect that is more prominent than that associated with complete deletion of the cytoplasmic tail. Separately, we did not investigate a potential role for integrin α6 (ITGA6), the only α integrin subunit with which ITGB4 associates. We speculate that ITGA6 may preferentially heterodimerize with overexpressed mutated ITGB4 in our experiments with subsequent consequences on EC inflammatory responses, an idea supported by evidence of a functional role for signaling by ITGA6 in other experimental models^25^,^26^. We recognize that ITGB4-mediated signaling is complex and the identification of relevant molecular targets remains to be fully investigated.

One potential target of interest is syndecan-1, a cell-surface proteoglycan that binds extraceullar matrix components but also binds the ITGB4 cytoplasmic domain^27^, is expressed by EC, and has been implicated as a mediator of ALI^28^. Moreover, we have observed markedly decreased syndecan-1 expression in simvastatin-treated EC (unpublished data) which we speculate may account for the attenuation of ITGB4 tyrosine phosphorylation despite significant increases in ITGB4 protein levels induced by simvastatin^7^,^29^. Further studies aimed at characterizing the role of syndecan-1 in EC inflammatory responses mediated by ITGB4 may further inform our current findings and, in turn, lead to additional insights into the elaboration and propagation of ALI clinically.

Finally, it should be noted that although our interest in ITGB4 is derived from our prior research focused on the potential role of statins in ALI, two randomized, controlled clinical trials of ALI patients were recently published which in fact reported no benefit with the administration of statins^30^,^31^. However, there were potential limitations to both studies, not the least of which was the administration of statins to patients relatively late in their clinical course. It may also be the case that while statins may have effects beneficial in ALI patients, effective therapeutic strategies may ultimately require more targeted approaches aimed at augmenting the specific statin effects most responsible for their benefits. If this is the case, our data support signaling mediated by ITGB4 as just such a target and warrants further investigation.

## Materials and Methods

### Cell culture

HPAEC cell lines were purchased from Lonza (Walkersville, MD) and were cultured in EGM-2 supplemented with 2% FBS, hydrocortisone, hFGF, VEGF, ascorbic acid, hEGF, GA-1000, heparin, and R3-IGF-1. Cells were incubated in 75 cm^2^ flasks and cultured at 37 °C in 5% CO_2_ and 95% air. All cells were used at passages 4–8.

### Materials and reagents

ITGB4 antibody (SC-9090) specific for the extracellular domain of ITGB4 was purchased from Santa Cruz Biotechnology (Santa Cruz, CA). A separate antibody specificially recognizing the ITGB4 cytosolic domain (LS-C14098) was purchased from Lifespan BioSciences (Seattle, WA). Human and mouse cytokines measurements were performed using commercially available kits as per the manufacturer’s instructions (Bio-Rad, Hercules, CA). All other antibodies and reagents were purchased from Santa Cruz Biotechnology (Santa Cruz, CA). ITGB4 mutated and deletion constructs were a generous gift from Dr. Filipo Giancotti (Memorial Sloan Kettering Cancer Center, New York, NY)^19^. Transgenically engineered mice expressing ITGB4 lacking the C-terminal signaling signaling segment of the ITGB4 tail were also provided by Dr. Giancotti’s lab. These animals express an ITGB4 that terminates at amino acid 1355, upstream of the four tyrosine phosphorylation sites^32^.

### Immunoprecipitation and Western blotting

For immunoprecipitation, cell lysates prepared from EC were incubated in immunoprecipitation buffer (50 mM HEPES (pH 7.5), 150 mM NaCl, 20 mM MgCl_2_, 1% Nonidet P-40, 0.4 mM Na_3_VO_4_, 40 mM NaF, 50 μM okadaic acid, 0.2 mM phenylmethylsulfonyl fluoride, and Calbiochem protease inhibitor mixture III at 1:250 dilution). The immunoprecipitation was carried with an anti-ITGB4 antibody.

For Western blotting, samples were prepared in RIPA buffer containing proteinase and phosphatase inhibitors as per standard protocols. After sonication and centrifugation, the supernatant was collected, Laemmli sample buffer added, and sampels were then boiled and analyzed by SDS–PAGE. After transfer to a nitrocellulose membrane (Bio-Rad, Inc., Hercules, CA), Western blotting was performed using appropriate primary antibodies and horseradish peroxidase-conjugated secondary antibodies prior to visualization via chemiluminescence (Amersham Biosciences, Piscataway, NJ). Blot density was determined by Alpha Imager software (Alpha Innotech, San Leandro, CA).

### Transfection of EC prior to mechanical stretch

Cells were plated (60–80% confluent) and transfected with a vector plasmid (PRK5) or specific ITGB4 plasmids using jetPRIME® transfection reagent (Polyplus Transfection, New York, NY). After incubating for 24 h, transfection efficiency of plasmids was verified by Western blotting. Of note, EC express relatively low levels of endogenous ITGB4 which was present in all cells in addition to the specific ITGB4 constructs transfected.

### Mechanical stretch of EC

EC were plated onto six-well silicone elastomer Bioflex plates coated with type I collagen (FlexCell International, Hillsborough, NC) and grown to 75–80% prior to transfection as described. Mechanical stretch was performed via the Flexcell Strain Unit (FX-3000; FlexCell International) placed in a 5% CO_2_ incubator at 37 °C and 95% humidity. The device uses a controlled vacuum to induce CS with 18% elongation at a frequency of 30 cycles per minute (0.5 Hz) for 6 h. The media was then collected and briefly centrifuged to measure cytokine levels with Bio-Rad Bio-Plex ELISA kits (Hercules, CA) according to the manufacturer’s instructions.

### Murine VILI model

All experiments and animal care procedures were approved by the University of Illinois at Chicago Animal Care and Use Committee and were performed in accordance with their guidelines and regulations. The ITGB4 mutant mice and wildtype C57/Bl6 mice, 8–12 weeks-old, were administered high tidal volume mechanical ventilation (V_T_ = 40 ml/kg, 65 breaths/min) for 4 h to induce VILI as we have previously described^33^. Mice were then sacrificed and bronchoalveolar lavage (BAL) was performed^3^. BAL fluid was assessed for total cell counts, protein content, and cytokine levels. Select mouse lungs were harvested for histological analysis.

### Statistical analysis

Student’s t-test was used to compare the means of data from two experimental groups while significant differences (p < 0.05) amongst multiple groups were confirmed by one-way ANOVA and Tukey’s *post hoc* multiple comparisons testing. Results are expressed as means ± SE.

## Additional Information

**How to cite this article**: Chen, W. *et al*. Role of Integrin β4 in Lung Endothelial Cell Inflammatory Responses to Mechanical Stress. *Sci. Rep.* **5**, 16529; doi: 10.1038/srep16529 (2015).

## References

[b1] Rubenfeld G. D. . Incidence and outcomes of acute lung injury. N. Engl. J. Med. 353, 1685–1693, (2005). 16236739 10.1056/NEJMoa050333

[b2] Ventilation with lower tidal volumes as compared with traditional tidal volumes for acute lung injury and the acute respiratory distress syndrome. The Acute Respiratory Distress Syndrome Network. *N. Engl. J. Med.* **342**, 1301–1308 (2000). 10.1056/NEJM200005043421801 10793162

[b3] Jacobson J. R. . Simvastatin attenuates vascular leak and inflammation in murine inflammatory lung injury. Am. J. Physiol. Lung Cell. Mol. Physiol. 288, L1026–1032 (2005). 15665042 10.1152/ajplung.00354.2004

[b4] Jacobson J. R. . Cytoskeletal activation and altered gene expression in endothelial barrier regulation by simvastatin. Am. J. Resp. Cell. Mol. Biol. 30, 662–670, (2004). 10.1165/rcmb.2003-0267OC 14630613

[b5] Chen W., Pendyala S., Natarajan V., Garcia J. G. & Jacobson J. R. Endothelial cell barrier protection by simvastatin: GTPase regulation and NADPH oxidase inhibition. Am. J. Physiol. Lung Cell. Mol. Physiol. 295, L575–583, (2008). 18658277 10.1152/ajplung.00428.2007 PMC2575942

[b6] Chen W. . Role of claudin-5 in the attenuation of murine acute lung injury by simvastatin. Am. J. Resp. Cell. Mol. Biol. 50, 328–336, (2014). 10.1165/rcmb.2013-0058OC PMC3930946 24028293

[b7] Chen W. . Critical role for integrin-beta4 in the attenuation of murine acute lung injury by simvastatin. *Am. J. Physiol. Lung. Cell. Mol. Physiol*. 303, L279–285, (2012). 22683568 10.1152/ajplung.00361.2011 PMC3423831

[b8] Chen W. . Integrin beta4 attenuates SHP-2 and MAPK signaling and reduces human lung endothelial inflammatory responses. J. Cell. Biochem. 110, 718–724, (2010). 20512931 10.1002/jcb.22582 PMC2879705

[b9] Ephstein Y. . Critical role of S1PR1 and integrin beta4 in HGF/c-Met-mediated increases in vascular integrity. J. Biol. Chem. 288, 2191–2200, (2013). 23212923 10.1074/jbc.M112.404780 PMC3554892

[b10] Su G. . Integrin alphavbeta5 regulates lung vascular permeability and pulmonary endothelial barrier function. Am. J. Resp. Cell. Mol. Biol. 36, 377–386, (2007). 10.1165/rcmb.2006-0238OC PMC1899321 17079779

[b11] Ganter M. T. . Interleukin-1beta causes acute lung injury via alphavbeta5 and alphavbeta6 integrin-dependent mechanisms. Circ. Res. 102, 804–812, (2008). 18276918 10.1161/CIRCRESAHA.107.161067 PMC2739091

[b12] Xu J. . Nonmuscle myosin light chain kinase mediates neutrophil transmigration in sepsis-induced lung inflammation by activating beta2 integrins. Nat.Immunol. 9, 880–886, (2008). 18587400 10.1038/ni.1628 PMC2553242

[b13] Su G. . Absence of integrin alphavbeta3 enhances vascular leak in mice by inhibiting endothelial cortical actin formation. Am. J. Resp. Crit. Care Med. 185, 58–66, (2012). 21980034 10.1164/rccm.201108-1381OC PMC3262039

[b14] Hogervorst F., Kuikman I., von dem Borne A. E. & Sonnenberg A. Cloning and sequence analysis of beta-4 cDNA: an integrin subunit that contains a unique 118 kd cytoplasmic domain. EMBO J. 9, 765–770 (1990). 2311578 10.1002/j.1460-2075.1990.tb08171.x PMC551734

[b15] Birukov K. G. . Magnitude-dependent regulation of pulmonary endothelial cell barrier function by cyclic stretch. *Am. J. Physiol. Lung. Cell. Mol. Physiol*. 285, L785–797, (2003). 12639843 10.1152/ajplung.00336.2002

[b16] Kariya Y. & Gu J. N-glycosylation of β4 integrin controls the adhesion and motility of keratinocytes. PLOS One 6, e27084, (2011). 22073258 10.1371/journal.pone.0027084 PMC3206902

[b17] Hemler M. E., Crouse C. & Sonnenberg A. Association of the VLA alpha 6 subunit with a novel protein. A possible alternative to the common VLA beta 1 subunit on certain cell lines. J. Biol. Chem. 264, 6529–6535 (1989). 2649503

[b18] Muller H. C. . Simvastatin attenuates ventilator-induced lung injury in mice. Crit. Care. 14, R143, (2010). 20673352 10.1186/cc9209 PMC2945124

[b19] Dans M. . Tyrosine phosphorylation of the beta 4 integrin cytoplasmic domain mediates Shc signaling to extracellular signal-regulated kinase and antagonizes formation of hemidesmosomes. J. Biol. Chem. 276, 1494–1502, (2001). 11044453 10.1074/jbc.M008663200

[b20] Ni X. . Interaction of integrin beta4 with S1P receptors in S1P- and HGF-induced endothelial barrier enhancement. J. Cell. Biochem. 115, 1187–1195 (2014). 24851274 10.1002/jcb.24770 PMC4374432

[b21] Singleton P. A. . CD44 regulates hepatocyte growth factor-mediated vascular integrity. Role of c-Met, Tiam1/Rac1, dynamin 2, and cortactin. J. Biol. Chem. 282, 30643–30657, (2007). 17702746 10.1074/jbc.M702573200

[b22] Giancotti F. G. Targeting integrin beta4 for cancer and anti-angiogenic therapy. Trends Pharmacol. Sci. 28, 506–511, (2007). 17822782 10.1016/j.tips.2007.08.004

[b23] Bertotti A., Comoglio P. M. & Trusolino L. Beta4 integrin activates a Shp2-Src signaling pathway that sustains HGF-induced anchorage-independent growth. J. Cell. Biol. 175, 993–1003, (2006). 17158954 10.1083/jcb.200605114 PMC2064708

[b24] Rabinovitz I., Tsomo L. & Mercurio A. M. Protein kinase C-alpha phosphorylation of specific serines in the connecting segment of the beta 4 integrin regulates the dynamics of type II hemidesmosomes. Mol. Cell. Biol. 24, 4351–4360 (2004). 15121854 10.1128/MCB.24.10.4351-4360.2004 PMC400463

[b25] Jiang R. & Grabel L. B. Function and differential regulation of the alpha 6 integrin isoforms during parietal endoderm differentiation. Exp. Cell. Res. 217, 195–204, (1995). 7698219 10.1006/excr.1995.1079

[b26] King T. E. . The role of alpha 6 integrin in prostate cancer migration and bone pain in a novel xenograft model. PLOS One 3, e3535, (2008). 18958175 10.1371/journal.pone.0003535 PMC2570216

[b27] Wang H., Jin H., Beauvais D. M. & Rapraeger A. C. Cytoplasmic domain interactions of syndecan-1 and syndecan-4 with alpha6beta4 integrin mediate human epidermal growth factor receptor (HER1 and HER2)-dependent motility and survival. J. Biol. Chem. 289, 30318–30332, (2014). 25202019 10.1074/jbc.M114.586438 PMC4215216

[b28] Li Q., Park P. W., Wilson C. L. & Parks W. C. Matrilysin shedding of syndecan-1 regulates chemokine mobilization and transepithelial efflux of neutrophils in acute lung injury. Cell 111, 635–646 (2002). 12464176 10.1016/s0092-8674(02)01079-6

[b29] Wang H., Leavitt L., Ramaswamy R. & Rapraeger A. C. Interaction of syndecan and alpha6beta4 integrin cytoplasmic domains: regulation of ErbB2-mediated integrin activation. J. Biol. Chem. 285, 13569–13579, (2010). 20181947 10.1074/jbc.M110.102137 PMC2859518

[b30] National Heart L. . Rosuvastatin for sepsis-associated acute respiratory distress syndrome. N. Engl. J. Med. 370, 2191–2200, (2014). 24835849 10.1056/NEJMoa1401520 PMC4241052

[b31] McAuley D. F. . Simvastatin in the acute respiratory distress syndrome. N. Engl. J. Med. 371, 1695–1703, (2014). 25268516 10.1056/NEJMoa1403285

[b32] Nikolopoulos S. N., Blaikie P., Yoshioka T., Guo W. & Giancotti F. G. Integrin beta4 signaling promotes tumor angiogenesis. Cancer Cell 6, 471–483, (2004). 15542431 10.1016/j.ccr.2004.09.029

[b33] Finigan J. H. . Activated protein C protects against ventilator-induced pulmonary capillary leak. Am. J. Physiol. Lung Cell. Mol. Physiol. 296, L1002–1011, (2009). 19363121 10.1152/ajplung.90555.2008 PMC2692806

